# Composition and Antioxidant Properties of Spanish Extra Virgin Olive Oil Regarding Cultivar, Harvest Year and Crop Stage

**DOI:** 10.3390/antiox8070217

**Published:** 2019-07-11

**Authors:** Thays Helena Borges, Adriana Serna, Luis Carlos López, Luis Lara, Rosa Nieto, Isabel Seiquer

**Affiliations:** 1 Departamento de Fisiología y Bioquímica de la Nutrición Animal, Estación Experimental del Zaidín, Consejo Superior de Investigaciones Científicas (CSIC), Camino del Jueves, Armilla, 18100 Granada, Spain; 2 Policlínica de Especialidades Médicas. Federico Medrano 620, San Francisco del Rincón, Guanajuato 36300, Mexico;; 3 Instituto de Biotecnología, Centro de Investigación Biomédica, Universidad de Granada, Avda. del Conocimiento, 18016 Granada, Spain

**Keywords:** extra virgin olive oil, Hojiblanca, Arbequina, antioxidant properties, polyphenols, chemometric analysis

## Abstract

The health benefits of extra virgin olive oil (EVOO) are related to its chemical composition and the presence of bioactive compounds with antioxidant properties. The aim of this study was to evaluate antioxidant compounds (pigments, coenzyme Q_10_ (CoQ_10_) and phenolic compounds) and antioxidant properties of EVOO from the same region comparing different cultivars (Hojiblanca and Arbequina), harvest year and crop stage. Antioxidant properties of oils were studied before and after a gastrointestinal digestion process, by in vitro assays (DPPH, ABTS and FRAP) and antioxidant markers in Caco-2 cells (reactive oxygen species production). The content of bioactive compounds measured was significantly affected by cultivar and harvest year (except for carotenoids) and by the crop stage (except for coenzyme Q_10_). Higher amounts of coenzyme Q_10_ were observed in Hojiblanca than in Arbequina EVOO. Total phenol content and antioxidant properties were also different depending on cultivar and harvest year and the in vitro digestion process strongly improved antioxidant marker values. Antioxidant potential in bioaccessible fractions was mainly related to the content of coenzyme Q_10_ and phenolic compounds in EVOO. Chemometric analysis showed that the oils were clearly classified by cultivars, harvest and crop stage, according to the chemical composition and antioxidant activity analyzed in the present study.

## 1. Introduction

The health benefits of extra virgin olive oil (EVOO) are significantly attributed to its high antioxidant potential, which, in turn, is deeply linked to its chemical composition [1]. Some of the bioactive compounds with antioxidant activity identified in EVOO are carotenoids, coenzymes and phenolic compounds, among others [2,3]. Multiple health benefits have been ascribed to phenolic compounds, such as prevention of cardiovascular disease; anti-inflamatory, antimicrobial and antiviral activities; and general protection against oxidative damage [4,5]. 

Antioxidants present in EVOO delay oxidative stress by inhibiting the formation or preventing the propagation of free radicals by several mechanisms; methods usually used to evaluate the antioxidant capacity are mainly focused on assessing the free radical scavenging ability, such as 2,2-azinobis (3-ethyl-benzothiazoline-6-sulfonic acid) (ABTS) and 2,2-diphenyl-1-picrylhydrazyl (DPPH) or the ferric reducing antioxidant power (FRAP) and are commonly applied in chemical extracts of the oils [6]. These methods may be useful to determine the oil oxidative stability of the oil, but the biological effect of antioxidant compounds in vivo will depend on their bioavailability [7]. In this sense, it is accepted that the primary requisite for a bioactive compound to exert an antioxidant activity in vivo is to be bioaccessible, i.e., be released from the from matrix during the digestive process and, moreover, maintain its properties after the biotransformations caused by the gastrointestinal digestion [8]. The determination of antioxidant activity after in vitro digestion, considering all the transformations of the EVOO matrix, and consequently of its bioactive compounds has been investigated in previous works [6,8,9,10,11].

It is widely known that composition and antioxidant properties of EVOO may be affected by several agronomic factors, such as cultivar, fruit ripening stage and agroclimatic conditions [5,12,13]. The olive ripeness and the cultivar have been described as the most important factors affecting the EVOO phenolic profile [14,15], while organoleptic characteristic such flavour have been linked to geographic and climatic factors more than to cultivar or ripening stage [13,16]. Nevertheless, studies about how these aspects could affect the bioaccessible fraction of EVOO are still very scarce.

In the present work, we aimed to evaluate some relevant aspects of chemical composition (pigments, coenzyme Q_10_ (CoQ_10_) and phenolic compounds) and the antioxidant potential of Spanish EVOO from the same geographic region, comparing different cultivars, harvest years and crop stages (early and late). As the content of bioactive compounds may also influence the oil colour, colour coordinates were also measured. The antioxidant activity was studied after and before an in vitro gastrointestinal digestion, and methods based on H-atom and single-electron transfer (DPPH, ABTS, FRAP) together with cell culture markers (generation of reactive oxygen species (ROS)) were applied. 

## 2. Materials and Methods

### 2.1. Chemicals

The chemicals used were all of high purity or analytical reagent grade. Bidistilled deionized water (Milli-Q purification system, Millipore, Bedford, MA, USA) was used. Ethanol and methanol were provided by VWR (Barcelona, Spain), sodium bicarbonate, acetate sodium, sodium carbonate, hydrochloric acid (37%), caffeic acid, hydrochloric acid, anhydrous sodium carbonate and potassium hexacyanoferrate (III) were acquired to Merck (Darmstad, Germany). Folin–Ciocalteau reagent, 6-hydroxy-2,5,7,8-tetramethyl-chroman-2-carboxylic acid (Trolox), DPPH, Pepsin, Pancreatin, bile salts, (4-(2-hydroxyethyl)-1-piperazineethanesulfonic acid) (HEPES) and tert-butylhydroperoxide (t-BOOH), the cell culture media, cell culture-grade chemicals, standards for individual phenolic compounds and. CoQ_10_ analysis were purchased to Sigma (Sigma-Aldrich, St, Louis, MO, USA). The ABTS was obtained from Amresco (Solon, USA). 2,4,6-Tri(2-pyridyl)-s-triazine (TPTZ) and iron (III) chloride for the ferric reducing power (FRAP) assay were from Fluka Chemicals (Fluka Chemicals, Madrid, Spain). 

### 2.2. Samples

EVOO from two cultivars (Arbequina and Hojiblanca) from the South of Spain (Estepa, Sevilla; latitude 30°17′N, longitude 4°52′W) were analyzed. The olives were harvested in two years (2014 and 2015) at two crop stages: early stage (September–October, stage 1) and late stage (November–December, stage 2). The oil was extracted within 24 hours under a two-phase extraction system. The samples (*n* = 3 from each stage and season) were directly donated by the producers from the same cooperative and sent to the Spanish National Research Council laboratories (CSIC, Granada, Spain) protected from light and high temperature until analysis. All the samples meet standards of quality established by the European Union regulation n° 2568/91 for EVOO The description of samples and the climatic conditions of seasons are presented in Table 1. 

### 2.3. Determination of CoQ_10_

The analysis of Co Q_10_ was performed as described by Borges et al. [17]. Briefly, 1-propanol was mixed with oil, vortexed and centrifuged at 11,300 *g*, 5 min. The supernatant was diluted (1/500) in 1-propanol before the high-performance liquid chromatography (HPLC) injection. CoQ_10_ in the oil extract was determined by HPLC (reversed-phase high-performance liquid chromatography, Gilson, Middleton, WI, USA) with a C18 symmetry column (3.5 μm, 4.6 × 150 mm) (Waters Chromatography, Barcelona, Spain) using methanol, ethanol, 2-propanol, glacial acetic acid (500:500:15:15) and 50 mM sodium acetate at a flow rate of 0.9 mL/min as a mobile phase. The electrochemical detector consisted of an ESA Coulochem III with the following setting: Guard cell (upstream of the injector) at +900 mV and the analytical cell at +350 mV. Reduced CoQ_10_ (ubiquinol) and oxidized CoQ_10_ (ubiquinone) could be determined by this method, although ubiquinol was not detected in our conditions of extraction and analysis. The CoQ_10_ concentration of the oxidized form was estimated comparing the peak areas with those of standard solutions; values of the calibration curve were reported previously [17]. The results were expressed in mg/L of sample.

### 2.4. Pigments (Chlorophylls and Carotenoids) and Colour

Chlorophylls and carotenoids were determined according to Minguez-Mosquera et al. [18]. The samples of oil were dissolved with cyclohexane (1.5:5 *w/v*) and the absorbance was measured with a UV spectrophotometer (Pharmaspec UV 1700, Shimadzu, Kyoto, Japan). The chlorophyll and the carotenoid fractions were determined at 670 and 470 nm, respectively. The results obtained are expressed as mg of pheophytin ‘‘a’’ and lutein per kg of oil, respectively.

Instrumental colour (CIE L*, a*, b*) was measured directly in the olive oil samples using a Minolta Colorimeter (CR-400, Konica Minolta Corp., Bremen, Germany) with illuminant D65, as described in Borges et al. [19]. 

### 2.5. Individual Phenolic Compounds

The individual phenolic compounds of the oil samples were determined after an extraction with methanol/water [80:20] according to the International Olive Oil Council [20]. The extracts were analyzed by UPLCTOF-MS following the method validated by Rivas et al. [21]. All the analytical parameters of the methods used are shown in Borges et al., [17].

### 2.6. In Vitro Digestion

The in vitro digestion was performed including sequential steps of gastric and intestinal digestion, as described by Borges et al. [6]. The EVOO samples mixed with Mili-Q water (1:10 *w/v*) were subjected to the two phases of digestion (2 h, 110 oscillations/min; 37 °C, each), gastric (pH 2, pepsin solution) and intestinal (pH 7, pancreatin/bile salts solution). The samples were always protected from light and were sonicated previously to each step. After centrifugation (10,000 rpm, 30 min, 4 °C, Sorvall RC 6 Plus centrifuge) the soluble or bioaccessible fraction (BF) was separated from the residual fraction (RF). Both fractions were stored at −80 °C in tubes protected from the light under a nitrogen blanket, until were used to analyze the total polyphenol content (TPC) and antioxidant activity. Aliquots of the BF were also used for assays in Caco-2 cells.

### 2.7. Total Phenolic Content and Antioxidant Activity

Previous to analysis of total phenolic content (TPC) and antioxidant activity, a chemical extraction was applied to the oil samples and the two fractions obtained after the in vitro digestion process (bioaccessible and residual fractions), following the procedure described by Borges et al. [11]. Samples were previously mixed with n-hexane and methanol/water (80:20 *v/v*) was used as extracting solvent.

The methodologies previously described by Borges et al. [6] were followed. A multilabel plate reader (Victor X3, Waltham, MA, USA) was used. TPC was determined by the Folin-Ciocalteu method and the absorbance was measured at 750 nm. The results were expressed in mg of caffeic acid equivalents (CAE) per kg of oil. The antioxidant capacity of the samples was studied following the ABTS and DPPH assays (for measuring the free radical scavenger activity) and the FRAP method (for assessing the reducing power). The final absorbance was measured at 750, 520 and 595 nm for ABTS, DPPH and FRAP assays, respectively. In each method a calibration curve of Trolox was performed and the results were expressed in mM of Trolox equivalents/kg of oil.

### 2.8. Cell Culture Assays

Caco-2 cells were purchased through the Cell Bank of Granada University (Granada, Spain) from the European Collection of Cell Cultures (ECACC). Culture flasks were provided by Corning Costar (Cambridge, MA, USA). The cells were maintained in 75 cm^2^ plastic flasks by serial passages, using as culture medium high-glucose Dulbecco’s modified minimal essential medium (DMEM), with heat-inactivated fetal bovine serum (FBS) (10%), NaHCO_3_ (3.7 g/L), nonessential amino acids (1%), HEPES (15 mM), bovine insulin (0.1 UI/mL) and 1% antibiotic-antimycotic solution. The cells were given fresh medium every 2–3 days and maintained under atmosphere of air/CO2 (95:5), 90% humidity and 37 °C.

The antioxidant potential of the BF from the digested oils was assessed at the cell level, by measuring the reactive oxygen species (ROS) generation. Determinations were carried out both at basal conditions and against an induced oxidative stress. Experiments were carried out with BF:FSB-free DMEM (1:2 *v/v*), as previous assay using the colorimetric MTT assay (3-(4,5-dime thylthiazol-2-yl)-2.5-diphenyltetrazolium bromide, Roche, Mannheim, Germany) showed that values of cell viability were never <85% at such conditions.

For determination of ROS generation we used the dichlorofluorescin (DCFH) assay as described by Seiquer et al. [8]. Briefly, cells were seeded in 24-well multiwell plates at 2 × 10^5^ cells/well and incubated at 37 °C for 48 h. After exposition to BF of oils (2 h), cells were treated with DCFH 100 lM and incubated for 1 h. The DCFH was removed and culture medium (for basal measurements) or t-BOOH 5 mM (to induce oxidation) was added to the wells. The absorbance was measured at a wavelength of 485 nm excitation and 535 nm emission, at 37 °C for 0–90 min. DFCH in the presence of ROS is converted into dichlorofluorescein (DCH) and emits fluorescence. The data were expressed as fluorescence units from at least two independent experiments (*n* = 3 per experiment).

### 2.9. Statistical Analyses

All data are presented as the means of three independent experiments. The data obtained were analyzed applying three-way analysis of variance (ANOVA), using cultivar, year and stage of harvest as independent variables. The differences were established at *P* < 0.05 and the interactions were evaluated. Tukey’s test was used to compare mean values between olive oils. In addition, chemometric analysis was performed including all the variables evaluated in the present study (minor compounds, colour coordinates and parameters related with antioxidant activity). Firstly, a hierarchical clustering analysis (HCA) was performed with the aim of find eventual similarities between the EVOO samples according with cultivar, year of harvest and crop stage, by calculating the multidimensional squared Euclidean distances of scores using the nearest neighbor method. In addition, to reduce the variables into a small number of factors and explore their impact in the oil differentiation, a factor analysis (FA) using a varimax rotation was applied. For all the statistical analysis the Stat Graphics Centurion XVI software (Stat Point Technologies, Inc., Dallas, TX, USA, 2013) was used.

## 3. Results and Discussion

### 3.1. Chemical Compounds and Colour

Table 2 shows the content of CoQ_10_, pigments (chlorophylls and carotenoids) and phenolic compounds, as well as the instrumental colour, determined in the EVOO. Phenolic compounds are presented grouped by families: flavonoids (mainly apigenin and luteolin), phenolic acids (mainly naringenin, p-coumaric acid, gallic acid and vanillic acid) and phenol alcohol (mainly hydroxytyrosol). The content of the individual polyphenols detected is provided as Supplementary Materials (sheet 1). 

Levels of CoQ_10_ were significantly affected by cultivar and harvest year, but crop stage did not have significant influence, except for Arbequina harvested in 2014. Comparing the varieties evaluated, Hojiblanca showed higher levels of CoQ_10_ than Arbequina (*P* < 0.001). Results are in accordance with a previous study [3] relating that Hojiblanca EVOO had higher levels than other commercial EVOOs from different geographic areas, including Arbequina, although with lower values than those found in the present study. Therefore, our results support that CoQ_10_ level in EVOO is mainly driven by genetic factors [3]. In addition, it may also be affected by climate and geographic conditions, and significant relationships with the altitude and the rainfalls of the growing areas have been found [17]. On the other hand, during the maturation process, the colour and composition of the olive fruit may change significantly and it has been suggested that EVOO harvested at early stages might be richer in CoQ levels [3]. On the contrary, we found that early or late crop stage did not affect CoQ_10_ content, and, as an exception, a positive effect of maturation was observed in Arbequina-2014 oils. Thus, it was confirmed that response to maturation is different depending on cultivar and may be also impacted by climatic conditions of the harvest year. Moreover, all the samples of the current study should be considered as very rich sources of CoQ_10_ (over than 50 mg/kg) according to the values previously established in the bibliography, which could contribute to the health benefits of the EVOO [22]. The high values of Hojiblanca-2015, over 200 mg CoQ_10_/L, must be highlighted among those found till the moment in the bibliography.

Regarding pigments, chlorophylls content was affected by cultivar, year and crop stage, whereas carotenoids were only impacted by stage of the harvest (*P* < 0.001). The highest levels of pigments were found in Arbequina-2015 from early stage, with 10.6 mg/kg and 5.35 mg/kg of chlorophylls and carotenoids, respectively, higher than those previously described for other virgin olive oil cultivars [23]. 

The ripening stage seemed to negatively affect pigment content, although the effect not was always significant. This influence was more consistently reported by Baccouri et al., [24] that found a significant loss of chlorophyll and carotenoids associated to the ripening degree in Tunisian monovarietal VOO. As ripening progresses in the olive fruit, photosynthetic activity decreases and the concentrations of both chlorophylls and carotenoids decrease progressively, whereas other coloured compounds, such as anthocyanins, are formed [25]. The presence of pigments has been also associated with the colour of virgin olive oils, which may vary from green-yellow to gold, depending on the variety and the stage of maturity [19,26]. It was observed that oils from the 2014 harvest were a more intense yellow (higher b*) than those from 2015, in both cultivars. Colour of oils plays an important role in the perceptions and preferences of consumers, which associate a dark green colour with high quality and pale yellow with refined and lower quality olive oils [27].

Flavonoids represented the majority group of the phenolic compounds determined in the present assay in all the EVOO analyzed, ranging from 42% of the total detected in Hojiblanca EVOO 2014, stage 2, to 91% in Arbequina 2014, stage 2. Levels of phenolic alcohols also varied widely, reaching proportions up to 42% of the total in EVOO from Arbequina 2015, at early stage. This behavior was similar to a previous study that described flavonoids as the major group of phenols in EVOO from Cobrançosa cultivar in different harvesting times [28]. In addition, in general, the values found in the present study were between the ranges of values related by Phenol Explorer [29]. According with our results, phenolic compounds of all the groups were significantly affected by the factors analyzed, i.e., cultivar, harvest year and crop stage (*P* < 0.001, Table 2). In agreement, it has been already observed that the polyphenols concentration of virgin olive oils varies greatly depending on the olive cultivar, agronomic practices and degree of fruit ripening, as well as on the conditions of processing (type of olive mill, malaxation, etc.) and fruit and oil storage [30]. Variations on phenolic composition of oils associated to olives ripening stage are caused by chemical reactions and activity of enzymes such as oxidoreductases, polyphenol oxidases and peroxidases [15]. It has been described that oxidation of phenolic compounds occurs with ripening, but the oxidation rate strongly depends on the cultivar, which may be related to the different distributions of the endogenous oxidoreductases in the pulp and the seed of the olive fruit [28]. In our assay, Hojiblanca oils showed higher phenol content than Arbequina for all harvest and crop stages, and a general increase in phenolic compounds was observed from early to late oils in the same harvest. Although higher levels of phenolic compounds have been usually found in early harvested oils compared with late harvested [31], a significant increase of some polyphenols, such as vanillin or *p*-coumaric acid, have also been described along fruit ripening [28], which should be in agreement with our findings. Thus, it is necessary to determine the best ripening stage for each variety, in order to obtain a high-quality olive oil.

### 3.2. Total Phenolic Content (TPC) and Antioxidant Activity Before and After In Vitro Digestion of Oils 

Results are shown in Table 3. The colorimetric assay of TPC, based on the reaction of the Folin–Ciocalteu reagent with the functional hydroxy groups of the phenolic compounds, was included as an easy and valid method for the quantification of total phenols [32]. Current bibliographic data show a large variation of phenols in EVOO samples, from a few to approximately 1200 mg/kg, depending on the cultivar and environmental variables, such as rainfalls and olives ripeness, among others [1]. In the present study, TPC content found in chemical extracts before digestion was higher in Hojiblanca (367−405 mg/kg) than in Arbequina (222−231 mg/kg) EVOO, but no effect of harvest year or crop stage was observed. In the same line, values of antioxidant activity (ABTS, DPPH and FRAP) were also increased in Hojiblanca oils compared with Arbequina samples (*P* < 0.01), showing than oils with more quantity of phenolic components had also higher ability of scavenging free radicals and reducing power. These results agree with previous information showing positive relationships between the phenolic content of a large number of EVOOs with their antioxidant properties [11,30]. However, the measured antioxidant markers varied greatly depending on the year of harvest and the crop stage, unlike what was observed for TPC. These findings support that antioxidant quality of EVOO could be also attributed to compounds other than polyphenols [11]. We propose that the high levels of CoQ_10_ of Hojibblanca-2015 EVOO have a positive role in its antioxidant power, as CoQ_10_ is an electron acceptor and a potent antioxidant [33]. Beside, chlorophylls display antioxidant activity in the dark, but they act as pro-oxidants in the light [27]. It was also shown that climatic conditions affect the antioxidant potential of oils, but in a different way depending on cultivars; thus, it was observed that Hojiblanca-2015 oils had higher values of ABTS and DPPH, and lower values of FRAP, than Hojiblanca-2014, but Arbequina oils behaved differently according to the harvest. Other authors have also found that the antioxidant activity of olive oils, measured by ABTS, decrease during ripening [34].

Analysis of the bioaccesible fractions obtained after the in vitro digestion of the oils showed increased values of TPC and antioxidant properties of all the samples compared with chemical extracts (Table 3), confirming the positive effects of the digestive process in the releasing of bioactive compounds previously observed in oils [5,8] and in other foods [35]. In addition, after digestion of oils it was found that TPC was not affected by cultivar, and free radical scavenging activity (ABTS and DPPH) was higher in Arbequina than in Hojiblanca bioaccessible fractions, on the contrary to that shown in chemical extracts. Thus, results support that the digestion process is essential in defining the antioxidant properties of oils, and changes produced during digestion should be considered to predict the healthy potential of oils in vivo. It has been shown that polyphenols from EVOO undergo extensive gastrointestinal biotransformation, producing various metabolites through hydrolysis or conjugation that retain or improve the potential beneficial effect of the original compounds [36]. Hydroxytyrosol presented increased recovery during the digestive process due to the hydrolysis of secoiridoid derivatives, and has been recognized as the most efficient free radical scavenger and radical chain breaker [5,36]. Thus, depending on the profile of bioactive compounds in the oils, the related antioxidant properties will evolve differently during the digestive process.

**Figure 1:**
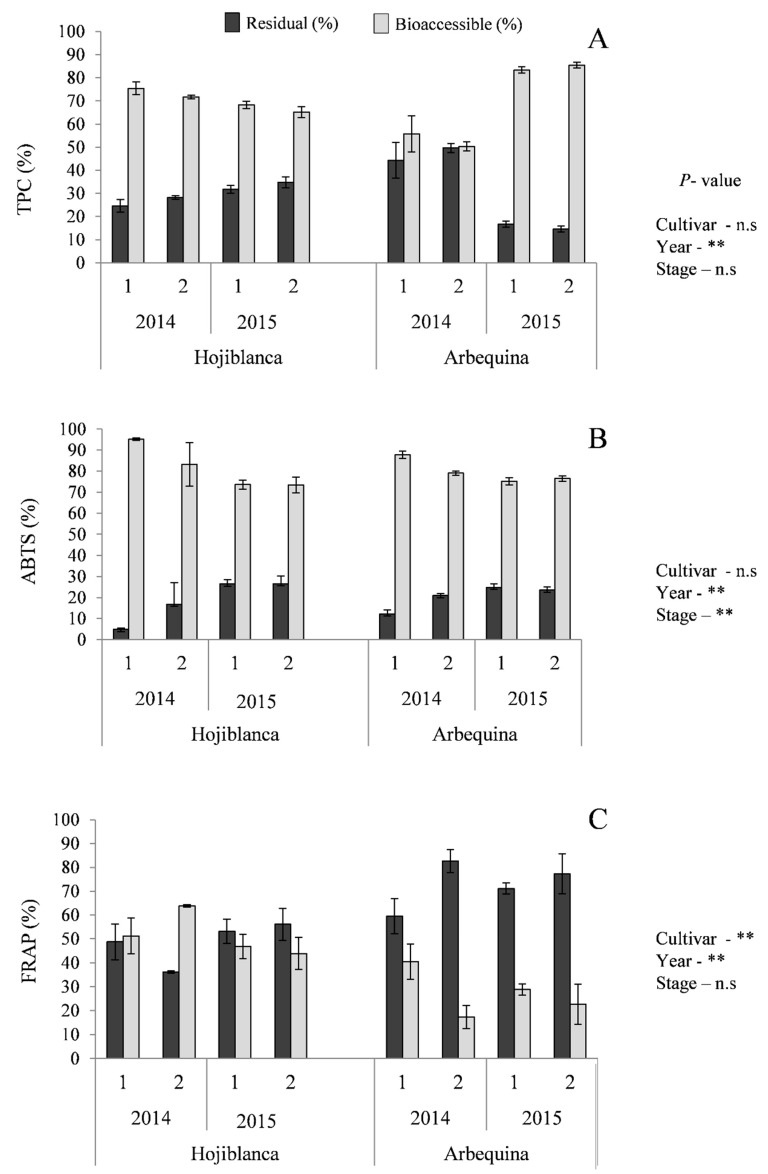
Average distribution of TPC (**A**) and antioxidant activity (ABTS, (**B**), and FRAP, (**C**)) evaluated after gastrointestinal digestion corresponding to residual (%) and bioaccessible (%) fractions. n.s. *P* > 0.05; ** *P* < 0.01.

Together with the soluble or bioaccessible fraction, a residual or non-soluble fraction after the in vitro digestion was also recovered and analyzed. This fraction is usually discarded when studying bioavailability of bioactive compounds, but it still may contain a large quantity of complexes which could be metabolically active, as it has been previously described after digestion of Arbequina EVOO of different origin [11]. According to our results, a range of 18−52% of TPC from the total determined after digestion was located in the residual part (Figure 1A). These compounds may act locally exerting an antioxidant and anti-inflammatory action at the intestinal level [37]. In agreement, this fraction contained substantial ABTS activity (5−25% from the total) and reducing power in higher proportions in some cases than the bioaccessible fraction (Figure 1B,C), although no DPPH activity was observed. These findings reveal a different distribution of individual antioxidant compounds between fractions: Compounds based in electron-transfer action (FRAP assay) were mainly located in residual parts after digestion, whereas bioaccessible fractions were enriched in H-atom transfer based antioxidants (ABTS assay). Moreover, polyphenols remaining in the large intestine after digestion may interact with the intestinal microbiota and be transformed into low molecular-weight structures which are potentially absorbable [38]. It was observed that cultivar in the present assay did not affect the distribution of TPC or ABTS activity in the different fractions obtained after digestion of the oils, although the year of harvest had a significant influence. 

**Figure 2:**
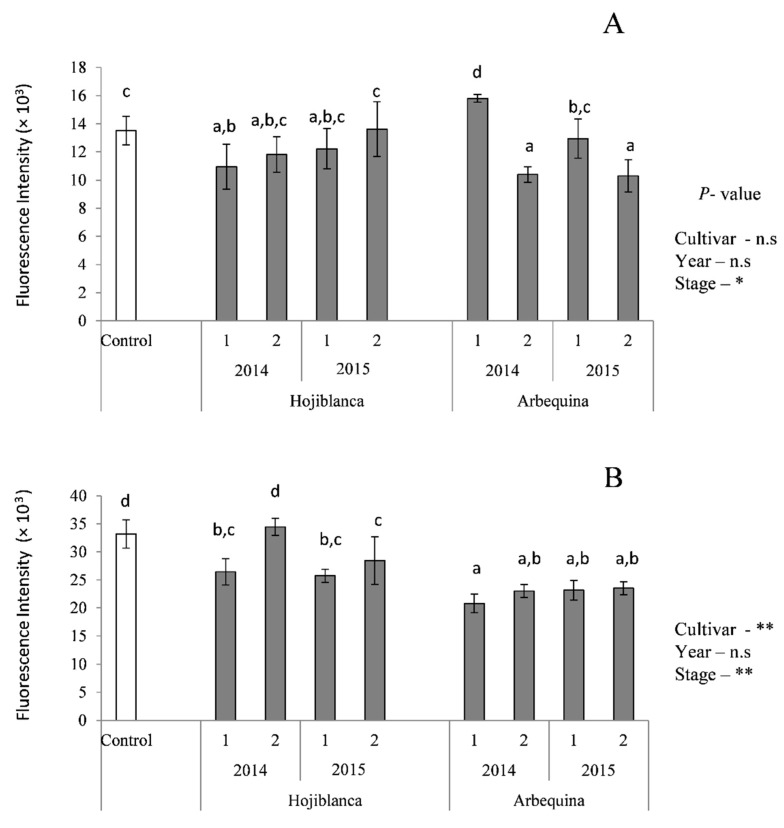
Reactive oxygen species (ROS) generation in Caco-2 cells expressed as fluorescence intensity at 90 minutes after incubation concerning the basal (**A**) and protective effect (**B**) oxidized with t-BOOH 5 mM. The control cells were incubated with culture medium only. n.s: *P* > 0.05; * *P* < 0.05; ** *P* < 0.01. Different letters indicate significant differences within samples and controls (*P* < 0.05).

Finally, the antioxidant activity was assessed by the ROS production in Caco-2 cells (Figure 2). After incubating the cells with the BF of the oils, a modest ROS generation was observed, in most cases similar to the of the control cells, and non-affected by cultivar or harvest year of the EVOO assayed (Figure 2A). When an oxidative injury is caused, preincubation of cells with oils over 2 h was able to protect by reducing ROS generation compared with control cells (with the only exception of Hojiblanca 2014, stage 2). In this case, Arbequina oils showed a higher protecting effect than Hojiblanca. It has been described that EVOO polyphenols and their active metabolites may reach a high enough concentration in the intestinal lumen to act as antioxidant and scavenge ROS [36]. The protectant effect of EVOOs against oxidative damage in Caco-2 cells has been mainly attributed to the most abundant phenolic compounds present, hydroxytyrosol, tyrosol and oleuropein [39]. Particularly hydroxytyrosol and its metabolites have shown an efficient role in protecting Caco-2 cells from the cytotoxic effects of oxidized low-density lipoprotein (LDL) and peroxyl radicals, due to their scavenging properties [40].

### 3.3. Chemometric Analysis

**Figure 3:**
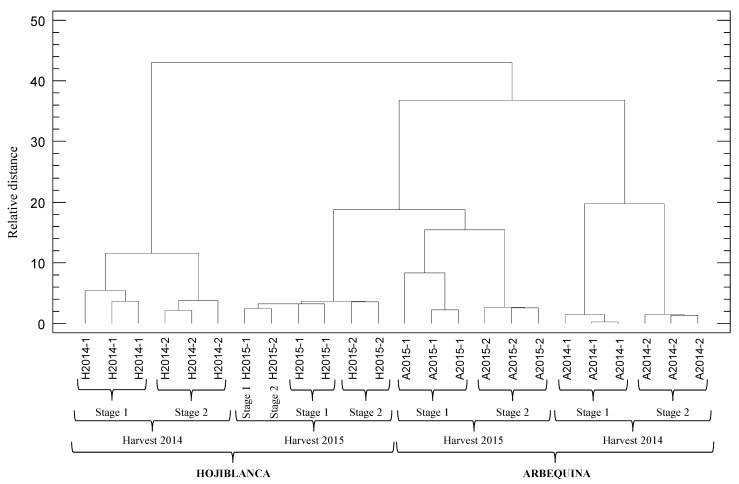
Dendogram generated using the nearest neighbor method and squared Euclidean distance measure, showing the conglomeration of EVOO samples obtained by clustering of all the variables analyzed in the present assay.

An HCA was initially applied for grouping samples that share common characteristics according to the analyzed variables and the dendogram obtained is shown in Figure 3. Four separated clusters were obtained by grouping the EVOOs by cultivar and year of harvest. According to the Euclidean distance, oils were clustered as follows: Hojiblanca-2015 < Hojiblanca-2014 < Arbequina-2015 < Arbequina-2014. In addition, all samples were well clustered by crop stage, with the exception of Hojiblanca-2015.

**Figure 4:**
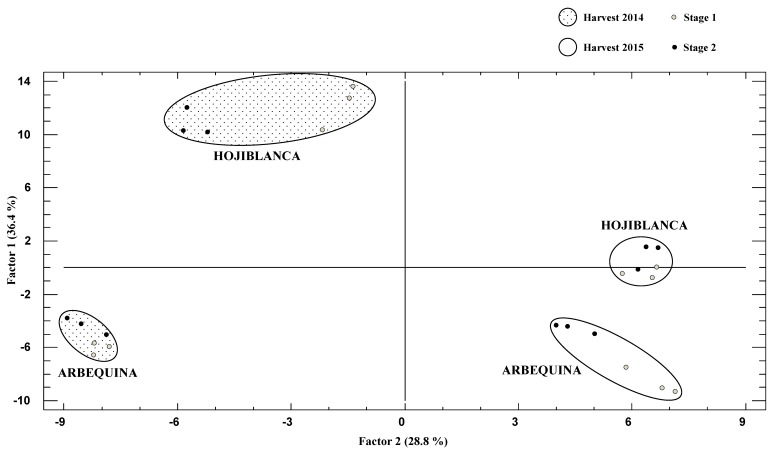
Factor analysis score graph of the two main factors (Factor 1 vs. Factor 2), considering all the variables analyzed in the EVOO from different cultivars, years and crop stage (*n* = 24).

In the FA, three factors justifying 79% of total variance were obtained (F1 36%, F2 29%, F3 14%). F1 was explained by antioxidant markers (FRAP in BF, chemical extracts and residual fractions, 0.92, 0.91 and 0.77, respectively, ROS protective effect 0.73), total phenol content in chemical extracts (0.87), phenolic acids (0.85), flavonoids (0.84) and chlorophylls (−0.78). F2 was mainly governed by colour (L*-0.96, b*-0.94, a*-0.84) and CoQ_10_ (0.88) and F3 was explained by DPPH in chemical extracts (−0.94) and carotenoids (0.84). The spatial representation of the oils according to F1 and F2 is depicted in Figure 4 and a clear separation of the samples according with cultivar and harvest year was observed (factor scores are provided as Supplementary Materials (sheet 2). Samples were separated by harvest year especially due to F2; oils from 2014 harvest were located in the left hand of the graph and those of 2015 in the right hand, according with their different colours and CoQ_10_ content. In addition, Hojiblanca cultivar oils were situated on the upper side (especially Hojiblanca-2014) whereas Arbequina samples were located in the lower side, mainly due to variables affecting F1, i.e., antioxidant properties, phenolic compounds and chlorophylls. Therefore, HCA was confirmed by the factor analysis, which in turn showed that the RVOO samples can be correctly classified according with the variables analyzed in the present assay. 

## 4. Conclusions

Findings of the present study confirm that composition and antioxidant properties of the EVOO strongly depend on cultivar and, within cultivars, also differ depending the year of harvest and the crop stage. The response of cultivars to climatic conditions may vary in each case and, as a consequence, different levels of bioactive compounds can increase or decrease during ripening. Antioxidant properties seem to be linked to phenolic content and profile, but other compounds, such as CoQ_10_, also have a significant role. Chemometric analysis showed that EVOOs may be classified by cultivars, harvest year and crop stage according the variables analyzed in the present assay. 

## Figures and Tables

**Table 1:** Description of the extra virgin olive oil (EVOO) samples.

Cultivar	Year	Stage	Mean Temperature (°C)	Mean Rainfall (mm)
Arbequina/Hojiblanca	2014	1	22.2	102.5
		2	16.2	140.7
Arbequina/Hojiblanca	2015	1	20.6	125.5
		2	16.2	45.2

Mean temperature and rainfall were supplied by the Spanish Meteoroly Agency, (Aemet, http://www.aemet.es), and corresponds to the province of Seville.

**Table 2:** Chemical compounds content (coenzyme Q_10_ (CoQ_10_), pigments and phenolic compounds) ^a^ and colour in EVOO.

Item	Hojiblanca				Arbequina				*P*-Value			
	2014		2015		2014		2015					
	1	2	1	2	1	2	1	2	SEM	Cultivar	Year	Stage
CoQ_10_ (mg/L)	152A	121A	206B	205B	57.4a	88.4b	138c	142c	2.28	**	**	n.s.
Chlorophylls (mg/kg)	2.24B	1.24A	3.58C	3.72C	3.66a	3.86a	10.6c	5.20b	0.06	**	**	**
Carotenoids (mg/kg)	4.28B	3.74B	2.10A	2.28A	1.86a	2.20ab	5.33c	2.53b	0.04	n.s.	n.s.	**
**Phenolic Compounds (μg/kg)**												
Flavonoids	1054C	1125D	816A	931B	508b	815c	343a	904d	0.51	**	**	**
Phenolic acids	274C	297D	116B	76A	28a	30a	55b	99c	0.51	**	**	**
Phenol alcohols	519C	640D	97A	135B	81b	55a	294c	464d	0.51	**	**	**
L*	22.6D	25.0C	19.3B	17.9A	26.4b	26.3b	18.0a	17.6a	0.21	**	**	n.s.
a*	0.53D	−0.79A	−0.04B	0.28C	−2.19a	−2.24a	0.24b	0.32b	0.01	**	**	**
b*	8.15D	12.3C	4.36B	2.55A	10.9b	12.5c	2.54a	2.46a	0.02	**	**	**

^a^ Results are expressed per L or kg of EVOO; Means within a line are mean values for each cultivar, year and crop stage. SEM: standard deviation of mean. Within each line, capital letters and lowercase represent statistical differences (*P* < 0.05) in each cultivar, Hojiblanca and Arbequina, respectively. n.s: *P* > 0.05; * *P* < 0.05; ** *P* < 0.01.

**Table 3:** Total phenolic content (TPC) and antioxidant activity (ABTS, DPPH and FRAP) determinate in the chemical extracts and bioaccesible fractions in EVOO.

Item	Hojiblanca				Arbequina				*P*-Value			
	2014		2015		2014		2015					
	1	2	1	2	1	2	1	2	SEM	Cultivar	Year	Stage
**Chemical Extracts**												
TPC	397A	367A	394A	405A	231a	222a	230a	226a	4.22	**	n.s.	n.s.
ABTS	0.69A	0.73B	0.80C	0.80C	0.73b	0.48a	0.79c	0.80 c	0.00	**	**	**
DPPH	0.67A	0.70A	1.39B	1.41B	1.52c	0.93b	0.70a	0.71a	0.01	**	**	**
FRAP	3.75C	3.44B	1.97A	2.10A	1.70C	0.98a	1.30b	1.36b	0.02	**	**	**
**Bioaccessible Fraction**												
TPC	1031B	633A	1018B	893B	371a	451a	1453b	1347b	15.8	n.s.	**	**
ABTS	3.90A	4.01A	4.41A	4.32A	4.33a	4.23a	4.37a	4.38a	0.04	**	**	n.s.
DPPH	0.98A	0.93A	4.97C	3.83B	2.28b	1.88a	5.44b	4.83b	0.06	**	**	**
FRAP	8.33B	7.77B	4.95A	4.95A	3.51a	2.98a	3.32a	2.86a	0.09	**	**	*

Total phenolic content (TPC) are expressed in mg of cafeic acid equivalents per kilogram of EVOO. Antioxidant activity (ABTS, DPPH and FRAP) are expressed in mmol of Trolox per kilogram of EVOO. Means within a line are mean values for each cultivar, year and crop stage. SEM: standard deviation of mean. Within each line, capital letters and lowercase represent statistical differences (*P* < 0.05) in each cultivar, Hojiblanca and Arbequina, respectively. n.s: *P* > 0.05; * *P* < 0.05; ** *P* < 0.01.

## Supplementary Materials

The following are available online at https://www.mdpi.com/2076-3921/8/7/217/s1.
